# Genetic and lifestyle factors for breast cancer risk assessment in Southeast China

**DOI:** 10.1002/cam4.6198

**Published:** 2023-06-02

**Authors:** Shuqing Zou, Yuxiang Lin, Xingxing Yu, Mikael Eriksson, Moufeng Lin, Fangmeng Fu, Haomin Yang

**Affiliations:** ^1^ Department of Epidemiology and Health Statistics, School of Public Health Fujian Medical University Fuzhou China; ^2^ Department of Breast Surgery Fujian Medical University Union Hospital Fuzhou China; ^3^ Department of General Surgery Fujian Medical University Union Hospital Fuzhou China; ^4^ Breast Cancer Institute, Fujian Medical University Fuzhou China; ^5^ Department of Medical Epidemiology and Biostatistics Karolinska Institutet Stockholm Sweden; ^6^ No.5 Hospital of Fuqing City Fuzhou China

**Keywords:** breast cancer, machine learning, risk assessment

## Abstract

**Background:**

Despite the rising incidence and mortality of breast cancer among women in China, there are currently few predictive models for breast cancer in the Chinese population and with low accuracy. This study aimed to identify major genetic and life‐style risk factors in a Chinese population for potential application in risk assessment models.

**Methods:**

A case–control study in southeast China was conducted including 1321 breast cancer patients and 2045 controls during 2013–2016, in which the data were randomly divided into a training set and a test set on a 7:3 scale. The association between genetic and life‐style factors and breast cancer was examined using logistic regression models. Using AUC curves, we also compared the performance of the logistic model to machine learning models, namely LASSO regression model and support vector machine (SVM), and the scores calculated from CKB, Gail and Tyrer–Cuzick models in the test set.

**Results:**

Among all factors considered, the best model was achieved when polygenetic risk score, lifestyle, and reproductive factors were considered jointly in the logistic regression model (AUC = 0.73; 95% CI: 0.70–0.77). The models created in this study performed better than those using scores calculated from the CKB, Gail, and Tyrer–Cuzick models. However, the logistic model and machine learning models did not significantly differ from one another.

**Conclusion:**

In summary, we have found genetic and lifestyle risk predictors for breast cancer with moderate discrimination, which might provide reference for breast cancer screening in southeast China. Further population‐based studies are needed to validate the model for future applications in personalized breast cancer screening programs.

## Graphical abstract

Among all factors considered, the best model was achieved when polygenetic risk score, lifestyle, and reproductive factors were considered jointly in the logistic regression model (AUC = 0.73; 95% CI: 0.70–0.77). The performance of the models established in this study is better than that using scores calculated from CKB, Gail and Tyrer–Cuzick models. However, there is no significant difference between the machine learning models and logistic model.

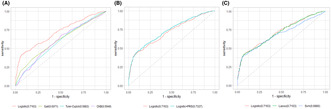

## 1 INTRODUCTION

Breast cancer is the most common type of cancer in women globally and the leading cause of cancer‐related deaths in women.^1^, ^2^ With 306,000 incident and 71,700 mortality cases in 2016, breast cancer continues to affect more women than any other type of cancer in China.^3^ Breast cancer risk may be predicted using the Gail, BRCAPRO, Tyrer–Cuzick, and Claus models, which have all been used extensively.^4^, ^5^, ^6^, ^7^, ^8^ However, the performances of models, are not consistent across study populations, which could indicate that effect estimates or risk factor distributions may vary across populations.^9^, ^10^

Many different factors contribute to breast cancer, including lifestyle risk factors and genetic factors.^11^, ^12^ Through genome‐wide association studies (GWAS), multiple genetic variants for breast cancer have been identified,^13^, ^14^, ^15^and turned into polygenic risk scores (PRS) to increase the precision of breast cancer prediction.^16^, ^17^ Several risk prediction models for breast cancer in China have been developed, which included merely lifestyle and reproductive factors.^18^, ^19^, ^20^ However, these models were based on small sample sizes, and therefore, the precision of validation estimates may not always be accurate. Additionally, single nucleotide polymorphisms (SNPs) were seldom included in models for predicting breast cancer risk, along with other traditional risk factors.^21^, ^22^, ^23^ Only one recent prospective study in China Kadoorie Biobank (CKB) showed acceptable calibration and moderate discrimination ability.^18^ Usage of hormone replacement treatment or a family history of breast cancer, however, were not taken into consideration by the model. Furthermore, risk factors for breast cancer may vary according to the estrogen receptor status.^24^ A recent research created PRS to estimate the probability of developing breast cancer and then further improved the model by stratifying analyses based on ER status.^25^

Machine learning techniques have been extensively employed to create risk assessment models in order to obtain more efficient and accurate decision‐making for the identification of breast cancer, including least absolute shrinkage and selection operator (LASSO), support vector machines (SVMs), artificial neural networks (ANNs), and Bayesian networks (BNs).^26^, ^27^, ^28^ However, the usability of various machine learning methods has yet to be verified externally. In recent years, LASSO has been increasingly employed in development of breast cancer risk assessment models, especially when more covariates are included in models.^29^ However, among previous studies in China, only one study in Hong Kong used a LASSO model to select risk factors,^20^ and no studies have compared the logistic regression model with the LASSO model.

In order to provide a reference for risk assessment, we evaluated breast cancer risk using several statistical models. In addition, the discriminatory performances of the models established in this study were compared with the scores calculated from CKB model, Gail model and Tyrer–Cuzick models.

## 2 MATERIALS AND METHODS

### 2.1 Study population

Women diagnosed with primary breast cancer at Fujian Medical University Union Hospital in southeast China between the ages of 18 and 90 years during the period from January 2013 to March 2016 were asked to participate in this study. The cancer diagnosis was provided and confirmed by two pathologists in Fujian Medical University Union Hospital, together with the information on estrogen receptor (ER) status and tumor stage. The healthy controls were selected from women who participated in opportunistic or organized health examinations in the same hospital during the corresponding period without history of cancer. We only collected information for those woman within age 18–90 years. From 2013 to 2016, a total of 1350 cases and 2053 controls were collected, after excluding participants with missing data, 1321 breast cancer patients and 2045 healthy controls were analyzed using a complete‐case approach. All the participants in this study were permanent residents in Fujian Province, southeast China. They answered a structured questionnaire during a face‐to‐face interview to gather comprehensive data on sociodemographic characteristics and possible breast cancer risk factors. The participants also donated blood for genotyping.

### 2.2 Risk factor selection and polygenic risk score

According to earlier researches on breast cancer risk factors in Chinese women's,^18^, ^19^, ^23^ the factors we selected for our study were age, body mass index (BMI), education level (primary school or lower, high school or higher), menopausal status (premenopausal, postmenopausal, and unnatural menopausal), age at menarche(≤13 years, 14–15 years, and ≥16 years), age at first live birth (≤24 years, 25–29 years, nonparous or ≥30 years), number of births (0, 1–2 and ≥3), breast feeding period (<12 months and ≥ 12 months), number of abortions (<2 and ≥2), oral contraceptives, hormone replacement therapy, family history of breast cancer and ovarian cancer (Yes vs. No), and prior breast surgery (Yes vs. No). These variables were also categorized according to the previous literature.^18^, ^19^, ^20^, ^21^, ^22^, ^23^ In addition, taking into account the low frequency use of oral contraceptive and hormone replacement therapy in our dataset, we combined these treatments into the use of exogenous hormones.

All participants' blood samples were genotyped using an SNPscan Kit (Genesky Biotechnologies) that was created specifically for this study. The kit was developed using double ligation and multiplex fluorescence PCR. From our earlier research^30^ and additional Chinese studies,^23^ three SNPs associated with breast cancer were chosen, including C6orf97 (rs2046210), CASC16 (rs129222061) and PRC1 (rs2290203). A weighted PRS was computed for each woman using the formula below:: PRS = *β*_1_ *x*_1_ + *β*_2_ *x*_2_ + … *β*_*k*_ *x*_*k*_ + *β*_*n*_ *x*_*n*_, where *β* is the per‐allele log odds ratio (OR) for the SNP_k_ risk allele for breast cancer, the number of alleles for a single SNP(0, 1, 2) is x_k_, while n is the total number of disease‐related SNPs in the profile. In Table S1, the SNPs and related log ORs (weights) that were utilized to derive the PRS are listed.

### 2.3 Statistical analyses

In this hospital‐based case–control study, participants were split into a training set and a test set at random in a 7:3 ratio, with 2356 participants in the training set and 1010 participants in the test set. The ideal model was created using the training set, and its performance was assessed using the test set. The distinctions between cases and controls in terms of general demographic and characteristic were assessed using the t‐test for continuous variables or *χ* ^2^ tests for categorical variables. We performed model development in the training set. A logistic regression model with only traditional risk factors, including age, BMI, education level, menopausal status, age at menarche, age at first live birth, number of births, breast feeding period, number of abortions, use of exogenous hormones, family history of breast cancer and ovarian cancer and prior breast surgery were used to examine associations with breast cancer risk. Our study also constructed a logistic regression model including both lifestyle risk factors and PRS in order to assess how genetic variation affects breast cancer collectively. Odds ratios (ORs) and their 95% confidence intervals (95% CI) were used to estimate the association between risk factors and breast cancer. In addition, we developed risk prediction models for ER+ and ER‐ breast cancer separately and compared them to the logistic regression model for breast cancer overall.

In order to enhance the model's performance, we also created machine learning models, namely the LASSO regression model and support vector machine (SVM), using gglasso^31^ and rminer^32^ packages, respectively. LASSO and SVM were previously used in the risk prediction for cancer, including breast cancer.^33^, ^34^, ^35^ Ten‐fold cross‐validation was applied for estimating coefficients used in LASSO regression. Coefficients in the LASSO regression were estimated by maximizing the log‐likelihood function. The regression performance of the SVM depends on the appropriate choice of parameter values. The SVM model is trained using the optimized parameters, and the validation set's performance is assessed using the improved parameters.

For the evaluation of the developed models, we used the test dataset. We estimated the breast cancer risk score using the CKB model, Gail model, and Tyrer–Cuzick model to compare their discrimination with the models developed in our study. The 5‐year CKB risk score was calculated based on the breast cancer risk prediction model developed using data from the China Kadoorie Biobank covering ten areas across China. This model takes into account factors like age, height, BMI, education, place of residence, age at menarche, parity, and family history of cancer.^18^ The Gail model includes age, age at menarche, age at first live birth, breast pathology, race and family history of breast cancer, while the Tyrer–Cuzick model further includes age at menopause, parity, hormone replacement therapy, BRAC mutation, and family history of ovarian cancer.^7^, ^36^ We used Breast Cancer Risk Assessment SAS Macro (Version 4, Gail Model) and IBIS command line program (v8.0b)^37^ to calculate Gail and Tyrer–Cuzick score, respectively. We tested the difference of AUC between our models and the logistic regression models using CKB score, Gail score or Tyrer–Cuzick score. Area under the receiver‐operating characteristic (ROC) curve (AUC) was used to assess performance. The delong test was used for pair‐wise comparison of AUC. Besides ROC and AUC, accuracy, Kappa value, sensitivity, and specificity were also used for the evaluation of the prediction models.

All the analyses were performed in R studio version 4.1.0. Statistical significance was determined using the threshold *p* < 0.05. All women gave their informed consent and the study was approval by the ethical committee in Fujian Medical University Union Hospital.

## 3 RESULTS

### 3.1 General demographic characteristics in the training set and test set

Table 1 provides a summary of the descriptive features of the training set (70%), which included 909 breast cancer patients and 1447 healthy controls, and the test set (30%), which included 412 breast cancer patients and 598 healthy controls. In both the training set and test set, breast cancer patients were more likely to have a higher level of education, earlier age at menarche, earlier age at first live birth, fewer number of births, shorter breast feeding period, more abortions, higher frequency of exogenous hormones use, previous benign breast disease diagnosis, and breast cancer family history. In addition, about 30% of the patients in this study were in the early stage (stage 0 + I) of breast cancer and nearly 70% of them were ER positive.

**TABLE 1:** Characteristics of breast cancer cases and health controls in training set and test set.

Characteristics	Training set				Test set			
	Cases (*n* = 909) (%)	Controls (*n* = 1447) (%)	Chi‐square	*p* Value	Cases (*n* = 412) (%)	Controls (*n* = 598) (%)	Chi‐square	*p* Value
Age (years) (mean ± SD)	47.1 ± 10.5	47.2 ± 10.7		0.967	46.9 ± 10.4	47.2 ± 10.6		0.662
Age (years)			1.085	0.781			1.135	0.769
18–40	258(28.4)	432(29.9)			119(28.9)	174(29.1)		
41–50	316(34.8)	490(33.9)			156(37.9)	209(34.9)		
51–60	236(26.0)	358(24.7)			96(23.3)	153(25.6)		
>61	99(10.8)	167(11.5)			41(9.9)	62(10.4)		
BMI (kg/m^2^) (mean ± SD)	22.7 ± 3.1	22.8 ± 3.1		0.193	22.5 ± 2.9	22.7 ± 2.9		0.336
Education level			5.518	0.019			7.318	0.007
Primary school or lower	343(37.7)	618(42.7)			148(35.9)	267(44.6)		
High school or more	566(62.3)	829(57.3)			264(64.1)	331(55.4)		
Menopausal status			1.956	0.375			2.918	0.233
Premenopausal	589(64.8)	897(62.0)			274(66.5)	375(62.8)		
Postmenopausal	283(31.1)	483(33.4)			118(28.6)	200(33.4)		
Unnatural menopausal	37(4.1)	67(4.6)			20(4.9)	23(3.8)		
Age at menarche (year)			18.223	<0.001			36.475	<0.001
≤ 13	202(22.2)	257(17.8)			102(24.8)	70(11.7)		
14–15	376(41.4)	538(37.2)			165(40.0)	230(38.5)		
≥ 16	331(36.4)	652(45.0)			145(35.2)	298(49.8)		
Age at first live birth (year)			21.523	<0.001			15.134	<0.001
<25	391(43.0)	754(52.1)			180(43.7)	323(54.0)		
25–29	390(42.9)	491(33.9)			162(39.3)	215(36.0)		
Nonparous or ≥30	128(14.1)	202(14.0)			70(17.0)	60(10.0)		
Number of births			31.429	<0.001			24.512	<0.001
0	41(4.5)	71(4.9)			28(6.8)	16(2.7)		
1–2	750(82.5)	1057(73.0)			332(80.6)	445(74.4)		
≥3	118(13.0)	319(22.1)			52(12.6)	137(22.9)		
Breastfeeding period(month)			18.727	<0.001			13.630	<0.001
<12	440(48.4)	568(39.3)			204(49.5)	225(37.6)		
≥12	469(51.6)	879(60.7)			208(50.5)	373(62.4)		
The number of abortion			258.590	<0.001			152.230	<0.001
<2	646(71.1)	1374(95.0)			280(68.0)	577(96.5)		
≥2	263(28.9)	73(5.0)			132(32.0)	21(3.5)		
Use of exogenous hormones			6.260	0.012			4.033	0.044
Yes	38(4.2)	33(2.3)			21(5.1)	15(2.5)		
No	871(95.8)	1414(97.7)			391(94.9)	583(97.5)		
Family history of breast cancer and ovarian cancer			45.885	<0.001			8.382	0.004
Yes	67(7.4)	25(1.7)			22(5.3)	11(1.8)		
No	842(92.6)	1422(98.3)			390(94.7)	587(98.2)		
Prior breast surgery			27.920	<0.001			31.035	<0.001
Yes	85(9.4)	57(3.9)			55(13.3)	22(3.7)		
No	824(90.6)	1390(96.1)			357(86.7)	576(96.3)		
Estrogen receptor (ER)								
Positive	636(70.0)				293(71.1)			
Negative	269(29.6)				116(28.2)			
Unknown	4(0.4)				3(0.7)			
Stage								
0	48(5.3)				21(5.1)			
I	233(25.6)				116(28.2)			
II	429(47.2)				176(42.7)			
III	172(18.9)				85(20.6)			
IV	4(0.5)				3(0.7)			
Unknow	23(2.5)				11(2.7)			

### 3.2 Results from the training set

For the training set, 12 variables were included in the backward logistic regression model, which ended up with 9 variables (Table 2). Age (OR = 1.01, 95% CI: 1.00–1.02) was linked to a higher risk of breast cancer. In addition, age at menarche ≥16 years old (OR = 0.77, 95% CI: 0.59–1.00) and parity ≥3 (OR = 0.53, 95% CI: 0.30–0.95) were inversely associated with breast cancer, while women with age at first live birth between 25 and 29 years old were more likely to get breast cancer (OR = 1.27, 95% CI: 1.03–1.58). For disease histories, family history of breast cancer and ovarian cancer, and prior breast surgery might increase the risk of breast cancer. Interestingly, number of abortions was strongly associated with breast cancer (OR = 7.13, 95% CI: 5.41–9.51) in our study.

**TABLE 2:** Logistic regression analyses on associations between established risk factors and breast cancer.

Characteristics	*β*	*P*	OR (95%CI)
Age	0.011	0.016	1.01 (1.00, 1.02)
Education level			
Primary school or lower			1.000 (REF)
High school or more	−0.160	0.142	0.85 (0.69, 1.05)
Age at menarche (year)			
≤13			1.000 (REF)
14–15	−0.042	0.741	0.96 (0.75, 1.23)
≥ 16	−0.262	0.052	0.77 (0.59, 1.00)
Age at first live birth (year)			
< 25			1.000 (REF)
25–29	0.241	0.029	1.27 (1.03, 1.58)
Nonparous or ≥30	0.096	0.579	1.10 (0.78, 1.54)
Number of births			
0			1.000 (REF)
1–2	−0.157	0.543	0.86 (0.52, 1.42)
≥3	−0.633	0.032	0.53 (0.30, 0.95)
The number of abortion			
<2			1.000 (REF)
≥2	1.965	<0.001	7.13 (5.41, 9.51)
Use of exogenous hormones			
No			1.000 (REF)
Yes	0.431	0.105	1.54 (0.91, 2.60)
Family history of breast cancer and ovarian cancer			
No			1.000 (REF)
Yes	1.448	<0.001	4.25 (2.62, 7.08)
Prior breast surgery			
No			1.000 (REF)
Yes	0.781	<0.001	2.18 (1.50, 3.18)

Similar to the results from the feature selection procedure in the backward logistic regression, LASSO regression model also selected age, age at menarche, age at first live birth, number of birth, number of abortion, family history and prior breast surgery as the main predictors for breast cancer. However, BMI and breast feeding period ≥12 months were associated with breast cancer in the LASSO model, but not involved in the backward logistic regression model. For SVM, all the 12 risk factors for breast cancer were included in the training procedure. Among the 12 variables, the top three important variables were abortion, family history of breast cancer and ovarian cancer and prior breast surgery (Table S2).

### 3.3 Comparing different models in the test set

**FIGURE 1:**
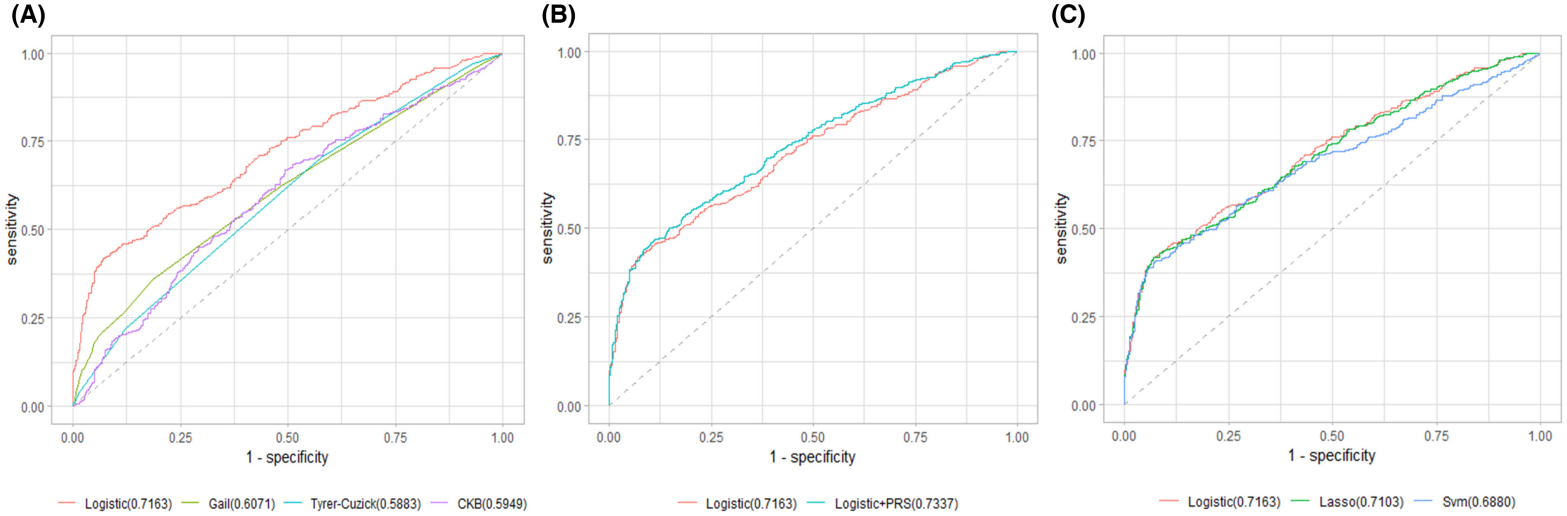
Receiver‐operating characteristic (ROC) curves for different risk assessment models of breast cancer. (A): ROC curves of logistic model that include only traditional risk factors compared with the models based on CKB score, Gail score and Tyrer–Cuzick score. (B): ROC curves of logistic model that include only traditional risk factors compared with logistics regression model that include both traditional risk factors and PRS. (C): ROC curves of logistic model that include only traditional risk factors compared with LASSO regression model and SVM m.

We examined the performance of various models we had created in the test set, as well as risk scores calculated from established breast cancer risk prediction tools (Table 3). The backward logistic regression model that included only traditional risk factors had an AUC of 0.72 (95% CI: 0.68–0.75, Sensitivity 41.7% and Specificity 93.0%) which was significantly better than the AUCs of models based on CKB score, Gail score and Tyrer–Cuzick score (Figure 1A). The discriminating ability of the model in our study can be marginally improved by adding PRS in the logistic regression model (AUC = 0.73, 95% CI: 0.70–0.77, *p* = 0.003, Figure 1B). The model established by LASSO (AUC = 0.71, 95% CI: 0.68–0.74) and SVM (AUC = 0.69, 95% CI: 0.65–0.72) showed no statistical difference as compared to the logistic regression model (Figure 1C). For the comparison of model performance, the LASSO model had the highest accuracy (72.18) and the logistic regression model that include both traditional risk factors and PRS model had the highest Kappa (0.382). However, taken together, the performance of the models developed in this study is similar (Table S3).

**TABLE 3:** The comparison of all the models in the test set.

Model^a^	AUC	95% CI	Sensitivity (%)	Specificity (%)	P^b^					
					Logistic (1)	Logistic + PRS (2)	Lasso (3)	SVM (4)	CKB (5)	Gail (6)
Logistic regression model that include only traditional risk factors (1)	0.7163	0.6836–0.7491	41.7	93.0						
Logistic regression model that include both traditional risk factors and PRS (2)	0.7337	0.7019–0.7655	46.1	89.8	0.003					
Lasso regression model (3)	0.7103	0.6773–0.7432	41.7	93.1	0.799	0.316				
SVM model (4)	0.6880	0.6533–0.7228	40.5	92.8	0.050	0.002	0.363			
CKB model (5)	0.5949	0.5593–0.6306	67.0	50.7	<0.001	<0.001	<0.001	<0.001		
Gail score (6)	0.6071	0.5728–0.6414	35.8	81.4	<0.001	<0.001	<0.001	0.001	0.630	
Tyrer–Cuzick score (7)	0.5883	0.5554–0.6213	70.4	42.3	<0.001	<0.001	<0.001	<0.001	0.791	0.476

^a^ Model 1: logistics regression model that includes only traditional risk factors; model 2: logistics regression model that includes both traditional risk factors and PRS; model 3: lasso regression model; model 4: SVM model; model 5: CKB model; model 6: Gail model; model 7: Tyrer–Cuzick model.

^b^ P (1) represents comparison with model 1, P (2) represents comparison with model 2, P (3) represents comparison with model3, P (4) represents comparison with model 4, P (5) represents comparison with model 5, P (6) represents comparison with model.

The 8 variables included in the model for ER+ breast cancer were age, age at menarche, age at first live birth, number of live birth, number of abortion, use of exogenous hormones, family history of breast cancer and ovarian cancer, and prior breast surgery, with an AUC of 0.73 (95% CI: 0.69–0.77, Sensitivity 41.7% and Specificity 92.8%). Only four factors, BMI, number of abortion, use of exogenous hormones and family history of breast cancer and ovarian cancer, were included in ER‐ breast cancer model, which had an AUC of 0.64 (95% CI: 0.57–0.72, Sensitivity 34.5% and Specificity 94.0%). However, there was no statistically statistical difference between the overall model and the risk prediction model stratified by ER status (Table S4).

## 4 DISCUSSION

In the current study, we created a moderate performing risk prediction model for breast cancer in southeast China. We confirmed that age, age at menarche, age at first live birth, number of births, abortions, family history of breast cancer and ovarian cancer and prior breast surgery are strong risk predictors for breast cancer. In addition, the performance of the model established in this study was better than models based on CKB score, Gail score and Tyrer–Cuzick score. However, the logistic model have no statistically significant difference between the LASSO model and SVM model. Our study also suggested that adding PRS can increase the predictive ability of the model.

Our study confirmed the association between several reproductive factors and breast cancer, including age at menarche, age at first live birth and number of births.^23^, ^38^ Breast development and function are directly impacted by hormones, and the risk of breast cancer is increased by reproductive variables linked to prolonged hormone exposure.^39^, ^40^, ^41^ Steroid hormones produced by the ovaries increase significantly during menarche, confirming an inverse association between later menarche age and breast cancer in our study.^42^ Estrogen and progesterone levels rise during pregnancy, and therefore age at first live birth and parity may influence breast cancer risk by influencing sex hormone levels.^43^ Using exogenous hormones may raise the chance of developing breast cancer. The risk of breast cancer increased considerably with long‐term contraceptive usage, but not with short‐term use, according to a meta‐analysis of 13 prospective cohort studies.^44^ Our study showed that use of exogenous hormones was not significantly associated with an increased risk of breast cancer in the multi‐variable adjusted model, probably because of the limited number of women using exogenous hormones in China.

Abortion is a substantial and persistent risk factor for breast cancer in Chinese women, as further evidenced by the current study. An earlier meta‐analysis in China found that when the rate of abortion rose, so did the risk of breast cancer.^45^ This association is biologically plausible as full‐term pregnancy is a protective factor for breast cancer, and the breast enlarges due to the changing level of estrogen and progesterone during pregnancy. Immature breast cells are more likely to transform into breast cancer cells when the pregnancy is ended via abortion, which raises the risk of breast cancer.^46^

With the best AUC values of 0.735 and 0.762, the Gail score and the Tyrer–Cuzick score are the two most popularly used breast cancer risk scores in American and European countries.^7^ The effectiveness of the Gail score in southeast China was limited according to our study, with a lower AUC compared with the developed logistic regression model. This limitation may be the result of the different effects of factors included in the Gail score between Asian and American women.^47^ In addition, the performance of the Tyrer–Cuzick score was not as good as the model we developed, probably because of the limited use of hormone replacement therapy in China and the missing information on BRAC mutation. These findings highlighted the value of employing a localized model for estimating the risk of breast cancer.

In China, several breast cancer risk assessment models have been developed based on data from other regions, with AUC varying from 0.573 to 0.658.^18^, ^19^, ^20^ A model created by Wang et al. that took into account family history of breast cancer, BMI, history of benign breast illness, and life satisfaction levels produced a C‐statistic of 0.64 in the test cohort.^19^ Han et al. found that the AUC from the CKB model was 0.658, while AUC from the externally validation study in Shanghai Women's Health Study was 0.573.^18^ Notably, the AUC of the logistic regression model developed in our study was over 0.70, which is considered acceptable performance. Our model was slightly better than these models, probably because of a better prediction performance when using localized training data.

Few models of breast cancer risk incorporate both non‐genetic and genetic components.^21^, ^22^, ^23^ In our study, the combination of traditional risk factors and PRS in the model increased the discrimination of the model, confirming recent findings based on 24 SNPs.^25^ These results suggest the use of genetic tests in future risk assessments for breast cancer.

SVM previously demonstrated the best performance when compared to other machine learning techniques for predicting breast cancer risk,^48^ and LASSO regression has been widely used in the selection of risk predictors. However, there was no statistically significant distinction between the models established by logistic regression, LASSO regression and SVM in our study. Interestingly, the SVM model had the highest accuracy and Kappa value among these models, indicating the need for more research on the use of machine learning algorithms for breast cancer risk prediction. More details on various risk variables may be required in order to improve the performance of these models because the effectiveness of machine learning methods depends on the features provided for the model.

Several studies have shown that developing risk models based on ER status of breast cancer may improve prediction and enable targeted screening and prevention in the future.^49^, ^50^ However, the risk prediction models stratified by ER status did not significantly improve the predictive ability of the model in our study, although the point estimates for AUC were slightly increased. This might be a result of reduced statistical power by stratified analysis.

This study has several advantages. Both genetic and non‐genetic factors were included in the model, which to some extend improved the power of the model. In addition, this study also applied machine learning methods, including LASSO and SVM, to compare different models. Other than the advantages mentioned above, this study has several limitations. First of all, this study is a hospital‐based case–control study with potential selection bias from the participants. We have a small proportion of patients with old age at diagnosis and late stage cancer. Therefore, our prediction model could not be generalized to these women. The reporting on the reproductive and familial risk factors could have been different between patients and controls due to recall bias. Further independent validation studies based on prospective cohorts are needed to test its accuracy and robustness. Thirdly, image‐based risk predictors for breast cancer such as mammographic density and micro‐calcification were not collected in current study, which might have reduced the performance of the model. BRAC mutations were not genotyped in our dataset, and therefore considered as missing in the calculation of Tyrer–Cuzick score, which might have reduced the performance of the model. However, as these variants are too rare in Chinese women, even in the cases,^51^ this missingness might not influence our results too much.

## 5 CONCLUSIONS

In summary, we confirmed that abortion, age, age at menarche, age at first live birth, number of births, family history of breast cancer and ovarian cancer and prior breast surgery are strong risk predictors for breast cancer. Our research also indicated that adding PRS might improve the model's capacity for risk prediction. These results can provide reference for personalized breast cancer screening in southeast China.

## ETHICAL APPROVAL

This study was approved by the ethics committee of Fujian Medical University Union Hospital (No. 2014021).

## CONSENT TO PARTICIPATE

Informed consent was obtained from all participants.

## AUTHOR CONTRIBUTIONS

**Shuqing Zou:** Data curation (equal); formal analysis (equal); investigation (equal); methodology (equal); writing – original draft (equal). **Yuxiang Lin:** Data curation (equal); formal analysis (equal); investigation (equal); methodology (equal); writing – review and editing (equal). **Xingxing Yu:** Data curation (equal); formal analysis (equal); investigation (equal); writing – review and editing (equal). **Mikael Eriksson:** Conceptualization (equal); resources (equal); supervision (equal); writing – review and editing (equal). **Moufeng Lin:** Data curation (equal); investigation (equal); writing – review and editing (equal). **Fangmeng Fu:** Funding acquisition (equal); project administration (equal); supervision (equal); writing – review and editing (equal). **Haomin Yang:** Funding acquisition (equal); project administration (equal); resources (equal); supervision (equal); writing – review and editing (equal).

## FUNDING INFORMATION

Haomin Yang is supported by the Natural Science Foundation of China [grant no: 82204132], Natural Science Foundation of Fujian Province [grant no: 2021 J01721], the Startup Fund for High‐level Talents of Fujian Medical University [grant no: XRCZX2020007], and Startup Fund for Scientific Research, Fujian Medical University [grant no: 2019QH1002]. The sponsor of the study had no role in the study design, data collection, data analysis, data interpretation, or writing of the report.

## Supporting information


Tables S1–S4

## Data Availability

Upon reasonable request and with the associated author's consent, data are accessible from the authors.
