# Supplementary material for: Genetic and lifestyle factors for breast cancer risk assessment in Southeast China
Source: Cancer Med. 2023 Jun 2;12(14):15504–14. doi: 10.1002/cam4.6198 (PMC10417168; doi:10.1002/cam4.6198)
Supplement: Supplementary file 1 — Tables S1–S4 [file CAM4-12-15504-s001.docx]

Supplementary Table 1 : Association between the SNPs and risk of breast cancer

| **SNP ID** | **Gene Name** | **Allele Risk** | **Chromosome position** | **OR(95%CI)** |
| --- | --- | --- | --- | --- |
| rs2046210 | C6orf97 | A/G | chr6:151627231 | 1.337(1.207,1.481) |
| rs12922061 | CASC16 | C/T | chr16:52601088 | 1.251(1.123,1.394) |
| rs2290203 | PRC1 | A/G | chr15:90968837 | 0.868(0.787,0.957) |

Supplementary Table 2: The importance of SVM model variables

| **Variable** | **Importance Value** |
| --- | --- |
| Age | 0.013 |
| BMI | 0.055 |
| Education level | 0.001 |
| Menopausal status | 0.016 |
| Age at menarche | 0.003 |
| Age at first live birth | 0.002 |
| Number of births | 0.008 |
| Breast feeding period | 0.004 |
| The number of abortion | 0.401 |
| Use of exogenous hormones | 0.001 |
| Family history of breast cancer and ovarian cancer | 0.327 |
| Prior breast surgery | 0.152 |

Supplementary Table 3: Performance evaluation for models

| Model | Accuracy(%) | Kappa | Sensitivity  （%） | Specificity （%） |
| --- | --- | --- | --- | --- |
| Logistic regression model that  include only traditional risk factors(1) | 72.08 | 0.375 | 41.7 | 93.0 |
| Logistic regression model that include both traditional risk factors and PRS(2) | 71.98 | 0.382 | 46.1 | 89.8 |
| Lasso regression model(3) | 72.18 | 0.377 | 41.7 | 93.1 |
| SVM model(4) | 71.49 | 0.361 | 40.5 | 92.8 |
| CKB model(5) | 57.33 | 0.167 | 67.0 | 50.7 |
| Gail score (6) | 62.83 | 0.183 | 35.8 | 81.4 |
| Tyrer-Cuzick score(7) | 53.76 | 0.117 | 70.4 | 42.3 |

Supplementary Table 4: Predictive performance of the ER+/ER− breast cancer

| Model | AUC | 95% CI | Sensitivity(%) | Specificity(%) | P |
| --- | --- | --- | --- | --- | --- |
|  |  |  |  |  | Logistic(1) |
| Logistic regression model that include only traditional risk factors(1) | 0.7163 | 0.6836-0.7491 | 41.7 | 93.0 |  |
| ER^+^(2) | 0.7309 | 0.6901-0.7716 | 41.7 | 92.8 | 0.586 |
| ER^-^(3) | 0.6445 | 0.5731-0.7160 | 34.5 | 94.0 | 0.074 |

^a^ model 1 represents logistics regression model that includes only traditional risk factors, model 2 represents ER^+^ breast cancer risk prediction model , model 3 represents ER^-^ breast cancer risk prediction model, ^b^ P (1) represents comparison with model 1, P (2) represents comparison with model 2.
